# Unprecedented nosocoimal spread os vancomycin-resistant *Enterococcus faecium* in a tertiary-care hospital in Switzerland

**DOI:** 10.1186/1753-6561-5-S6-P22

**Published:** 2011-06-29

**Authors:** L Senn, C Petignat, G Prod'hom, G Greub, P Basset, DS Blanc, G Zanetti

**Affiliations:** 1 CHUV, Lausanne, Switzerland

## Introduction / objectives

The incidence of vancomycin-resistant enterococci (VRE) remains sporadic in Switzerland. We report an unprecedented VRE (*E.faecium* van B) outbreak in a 900-bed tertiary care hospital and describe its molecular epidemiology.

## Methods

VRE was detected in clinical specimens by standard procedures. Carriage screening was performed by rectal swab. Swabs were inoculated into an enrichment broth and grown on chromogenic VRE agar. Isolates were typed by PFGE.

## Results

In November 2010, a first case of VRE was detected in a urine culture. The investigation identified 3 secondary cases in roommates (asymptomatic carriage). A second clinical case was detected in January 2011. Four secondary cases were identified. Both index cases were previously hospitalized in the same regional hospital. All patients transferred from the regional hospital were screened for VRE. In addition, weekly screening was initiated in patients hospitalized in the epidemic ward.

In total, from November 2010 to March 2011, 31 VRE cases were identified: 4 in clinical specimens (urine 2, wound 2) and 27 in screening swabs. One patient presented a VRE bacteremia. Molecular analysis showed that all isolates except 2 had the same PFGE pattern. The 2 different strains had only a variation of 1 to 2 bands and were probably related to the outbreak. Infection control measures (contact isolation of carriers and roommates, quarantine of the epdiemic ward and reinforcement of environment disinfection) were implemented.

## Conclusion

This the first report of a large VRE outbreak in Switzerland. Molecular typing confirmed the occurrence of an outbreak and showed that no sporadic case occurred during this period. The outbreak was controlled by implementation of strict infection control measures.

## Disclosure of interest

None declared.

